# Supplementary material for: Impact of Data and Study Characteristics on Microbiome Volatility Estimates
Source: Genes (Basel). 2023 Jan 14;14(1):218. doi: 10.3390/genes14010218 (PMC9859452; doi:10.3390/genes14010218)
Supplement: Supplementary file 1 [file genes-14-00218-s001.zip › genes-2109812-supplementary.pdf]

Impact of Data and Study Characteristics on Microbiome Volatility Estimates

Supplemental Information

Supplemental Methods

Approach to Subsampling

1. Start with the sample at the earliest time point.
2. Identify current sample as the start of the time interval.
3. Find the end point of the interval:
   a) Check whether a sample exists at exactly the desired sampling interval. If so, identify that sample as the end point of the interval.
   b) Else, check whether a sample exists within the acceptable window. If so, identify sample closest to the desired time point as the end point of the interval. Preference is given to shorter windows/earlier time points if there are ties.
   c) Else, move start of interval to the next observed time point. Iterate until a satisfactory interval is found.
4. Identify the end of previous successful interval as the start of the next interval.
5. Iterate #3 and #4 until the end of the time series.

Supplemental Figures and Tables

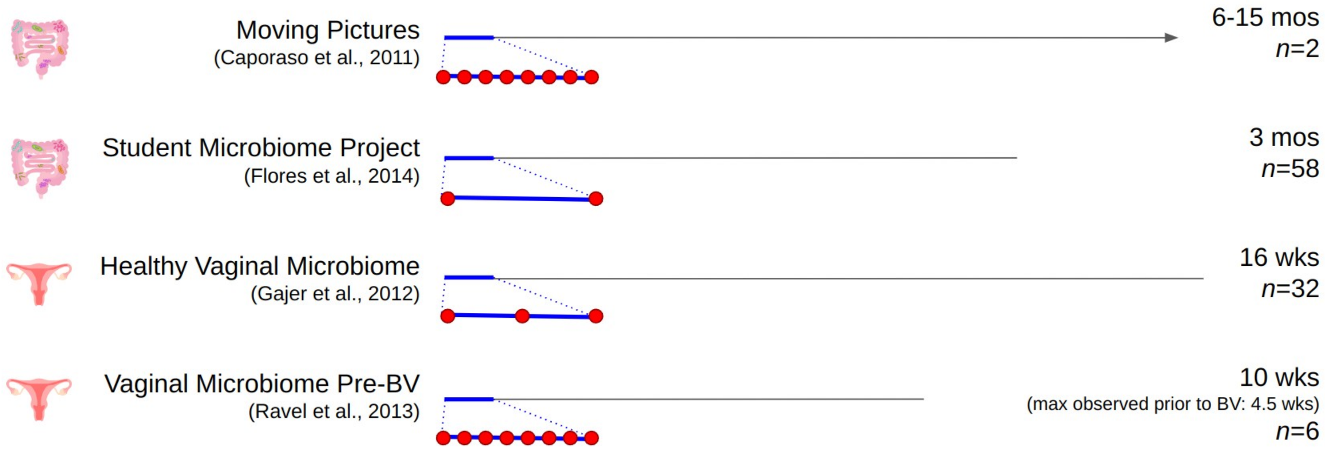

Figure S1. Selected data and study characteristics for the four studies used in this investigation. Call-out segments correspond to one week of each study, within which sampling frequency is indicated by red circles.

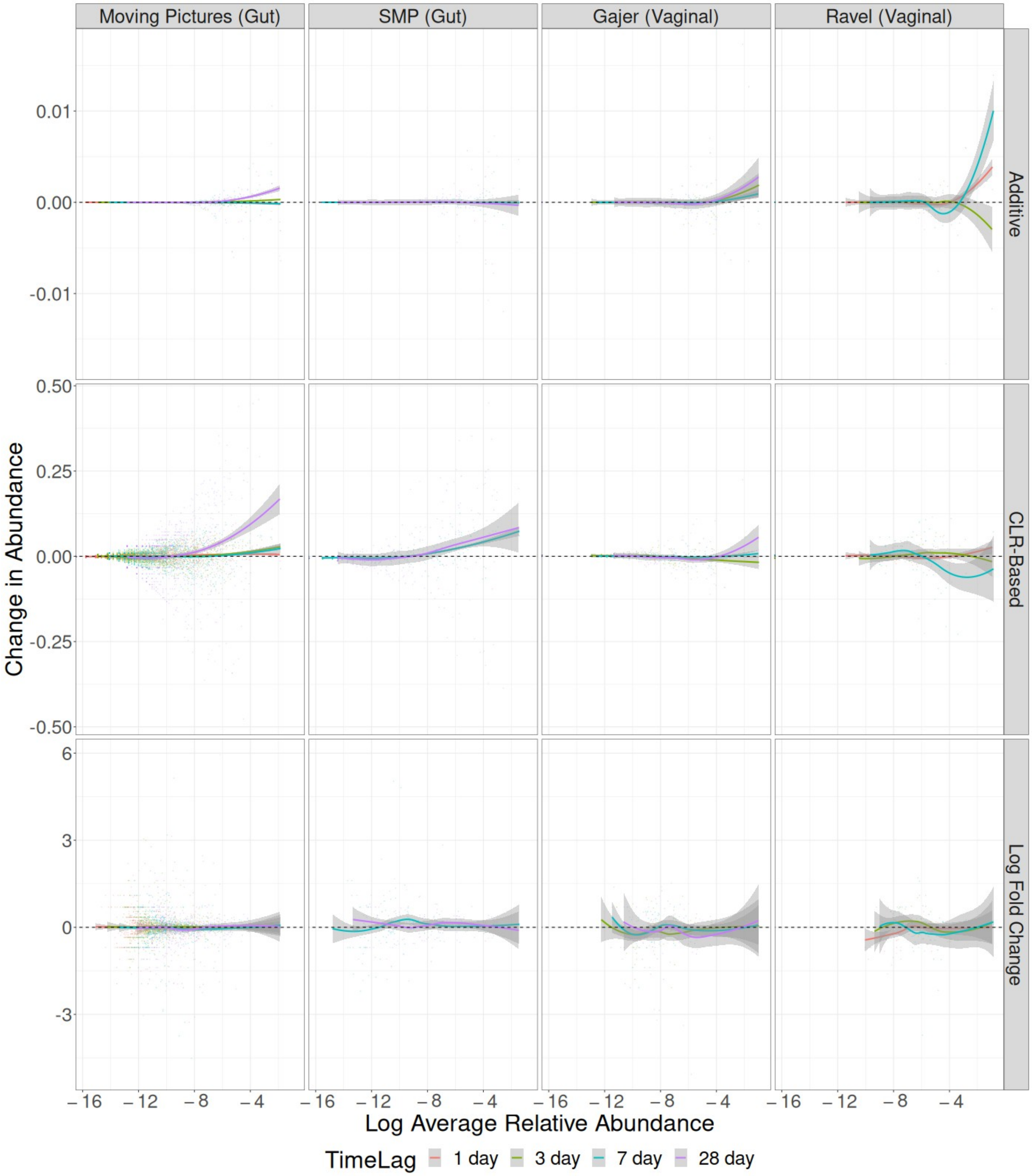

Figure S2. Average change in abundance for each taxon at each sampling interval vs. the taxon's relative abundance (small points), with overlaid LOESS smoothers. Changes in taxon abundance are generally centered around 0 regardless of sampling interval and the taxon's average relative abundance. The LOESS curves for the Ravel study show more variability around zero due to the smaller number of nonzero pairs.

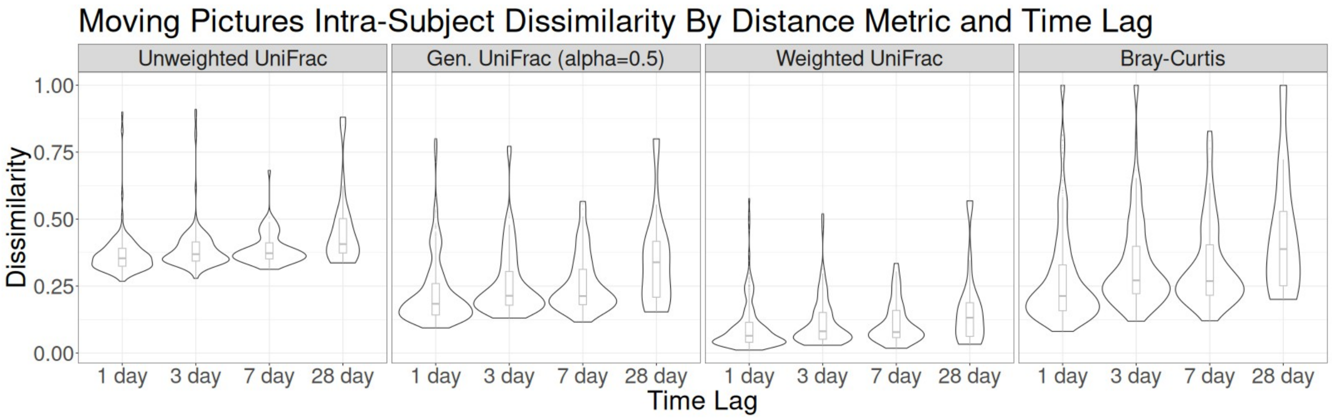

Figure S3. Intraindividual dissimilarity quantified using four metrics (unweighted UniFrac, generalized UniFrac, weighted UniFrac, and Bray-Curtis dissimilarity) for each time lag in the Moving Pictures study.

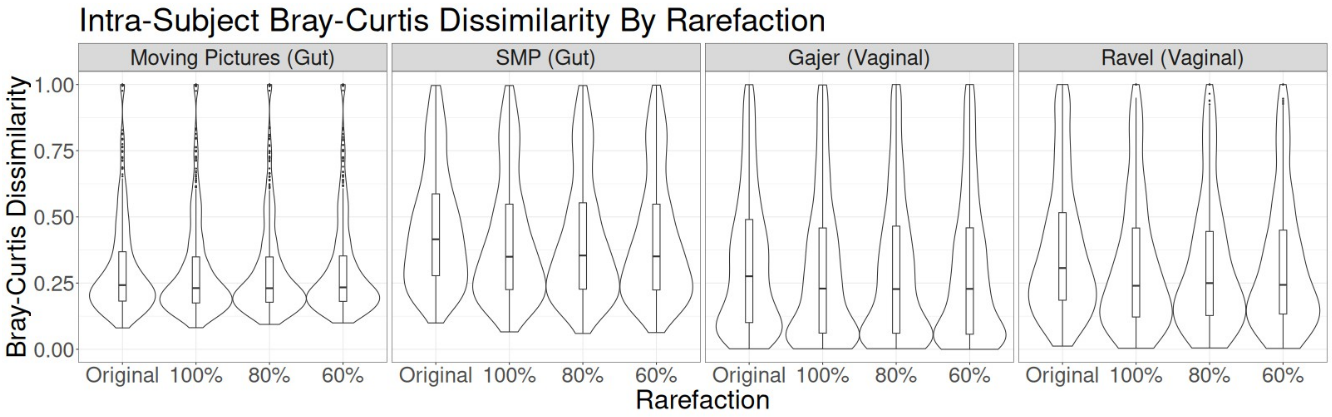

Figure S4. Distribution of intraindividual Bray-Curtis dissimilarity across rarefaction approaches for each of the four studies.

Table S1. Standard deviation (n pairs) of additive changes in taxon relative abundance by study, time lag, and average relative abundance category for each taxon.

		**Time Lag**			
**Study**	**Taxon Abundance**	**1 day**	**3 day**	**7 day**	**28 day**
**Moving Pictures (Gut)**	**All**	**0.00246 (1,549,142)**	**0.00269 (629,958)**	**0.00290 (309,036)**	**0.00407 (87,164)**
	[0, 10^{-05}]	2.2×10^{-05} (1,200,370)	2.0×10^{-05} (487,494)	1.5×10^{-05} (241,176)	1.3×10^{-05} (66,792)
	(10^{-05}, 10^{-04}]	0.000279 (209,967)	0.000231 (86,496)	0.000142 (41,574)	0.000147 (12,738)
	(10^{-04}, 10^{-03}]	0.00219 (96,577)	0.00200 (38,319)	0.00128 (18,330)	0.00136 (5236)
	(10^{-03} ,1]	0.0145 (42,228)	0.0158 (17,649)	0.0180 (7956)	0.0245 (2398)
**Student Microbiome Project (Gut)**	**All**	**-**	**-**	**0.0132 (234,472)**	**0.0140 (65,096)**
	[0, 10^{-05}]	-	-	3.2×10^{-05} (171,031)	1.9×10^{-05} (47,895)
	(10^{-05}, 10^{-04}]	-	-	0.000606 (24,486)	0.000355 (6283)
	(10^{-04}, 10^{-03}]	-	-	0.00555 (16,695)	0.00364 (4326)
	(10^{-03} ,1]	-	-	0.0427 (22,260)	0.0439 (6592)
**Gajer (Vaginal)**	**All**	**-**	**0.0208 (124,080)**	**0.0240 (147,510)**	**0.0285 (39,270)**
	[0, 10^{-05}]	-	9.5×10^{-05} (57,904)	7.4×10^{-05} (71,073)	0 (18,921)
	(10^{-05}, 10^{-04}]	-	0.000739 (29,328)	0.000629 (33,525)	0.00052 (9639)
	(10^{-04}, 10^{-03}]	-	0.00813 (23,312)	0.00542 (26,373)	0.00396 (6069)
	(10^{-03} ,1]	-	0.0620 (13,536)	0.0714 (16,539)	0.0826 (4641)
**Ravel (Vaginal)**	**All**	**0.0316 (25,670)**	**0.0374 (9211)**	**0.0369 (3926)**	**-**
	[0, 10^{-05}]	0 (6290)	0 (3050)	0 (1664)	-
	(10^{-05}, 10^{-04}]	0.000522 (4760)	0.000551 (976)	0.000493 (182)	-
	(10^{-04}, 10^{-03}]	0.00265 (6290)	0.00317 (2318)	0.00240 (1040)	-
	(10^{-03} ,1]	0.0554 (8330)	0.0669 (2867)	0.0716 (1040)	-

Table S2. Standard deviation (n pairs) of multiplicative changes between consecutive nonzero relative abundances by study, time lag, and average relative abundance category for each taxon.

		**Time Lag**			
**Study**	**Taxon Abundance**	**1 day**	**3 day**	**7 day**	**28 day**
**Moving Pictures (Gut)**	**All**	**1.00 (99,531)**	**1.09 (39,530)**	**1.12 (19,317)**	**1.32 (4582)**
	$[0, 10^{-05}]$	0.54 (2438)	0.53 (873)	0.45 (379)	0.29 (34)
	$(10^{-05}, 10^{-04}]$	0.80 (23,824)	0.84 (9424)	0.83 (4621)	0.87 (1088)
	$(10^{-04,}, 10^{-03}]$	1.02 (43,397)	1.11 (17,071)	1.15 (8486)	1.26 (2018)
	$(10^{-03}, 1]$	1.15 (29,872)	1.26 (12,162)	1.30 (5831)	1.66 (1442)
**Student Microbiome Project (Gut)**	**All**	**-**	**-**	**1.68 (17,074)**	**1.85 (4619)**
	$[0, 10^{-05}]$	-	-	0.97 (109)	0.61 (32)
	$(10^{-05}, 10^{-04}]$	-	-	1.30 (723)	1.17 (186)
	$(10^{-04,}, 10^{-03}]$	-	-	1.48 (2801)	1.61 (615)
	$(10^{-03}, 1]$	-	-	1.74 (13,441)	1.92 (3786)
**Gajer (Vaginal)**	**All**	**-**	**1.36 (2813)**	**1.52 (3112)**	**1.62 (813)**
	$[0, 10^{-05}]$	-	0.35 (4)	NA (0)	NA (0)
	$(10^{-05}, 10^{-04}]$	-	0.89 (72)	0.80 (34)	0.37 (11)
	$(10^{-04,}, 10^{-03}]$	-	1.29 (438)	1.23 (467)	1.26 (109)
	$(10^{-03}, 1]$	-	1.38 (2299)	1.57 (2611)	1.68 (693)
**Ravel (Vaginal)**	**All**	**1.08 (2103)**	**1.24 (691)**	**1.40 (264)**	**-**
	$[0, 10^{-05}]$	NA (0)	NA (0)	NA (0)	-
	$(10^{-05}, 10^{-04}]$	0.78 (7)	0 (2)	NA (0)	-
	$(10^{-04,}, 10^{-03}]$	0.97 (175)	0.77 (53)	0.83 (22)	-
	$(10^{-03}, 1]$	1.09 (1921)	1.27 (636)	1.44 (242)	-

Table S3. Standard deviation (n pairs) of differences in CLR-transformed abundances between consecutive time points by study, time lag, and average relative abundance category for each taxon.

		Time Lag			
**Study**	**Taxon Abundance**	**1 day**	**3 day**	**7 day**	**28 day**
**Moving Pictures (Gut)**	**All**	**0.37 (1,549,142)**	**0.41 (629,958)**	**0.40 (309,036)**	**0.51 (87,164)**
	$[0, 10^{-05}]$	0.14 (1,200,370)	0.14 (487,494)	0.13 (241,176)	0.14 (66,792)
	$(10^{-05}, 10^{-04}]$	0.52 (209,967)	0.55 (86,496)	0.56 (41,574)	0.62 (12,738)
	$(10^{-04}, 10^{-03}]$	0.87 (96,577)	0.96 (38,319)	0.99 (18,330)	1.23 (5236)
	$(10^{-03}, 1]$	1.19 (42,228)	1.34 (17,649)	1.29 (7956)	1.94 (2398)
**Student Microbiome Project (Gut)**	**All**	**-**	**-**	**0.57 (234,472)**	**0.64 (65,096)**
	$[0, 10^{-05}]$	-	-	0.11 (171,031)	0.12 (47,895)
	$(10^{-05}, 10^{-04}]$	-	-	0.47 (24,486)	0.49 (6283)
	$(10^{-04}, 10^{-03}]$	-	-	0.91 (16,695)	0.97 (4326)
	$(10^{-03}, 1]$	-	-	1.57 (22,260)	1.77 (6592)
**Gajer (Vaginal)**	**All**	**-**	**0.31 (124,080)**	**0.35 (147,510)**	**0.38 (39,270)**
	$[0, 10^{-05}]$	-	0.04 (57,904)	0.05 (71,073)	0.04 (18,921)
	$(10^{-05}, 10^{-04}]$	-	0.13 (29,328)	0.13 (33,525)	0.14 (9639)
	$(10^{-04}, 10^{-03}]$	-	0.32 (23,312)	0.33 (26,373)	0.36 (6069)
	$(10^{-03}, 1]$	-	0.8 (13,536)	0.92 (16,539)	0.99 (4641)
**Ravel (Vaginal)**	**All**	**0.47 (25,670)**	**0.55 (9211)**	**0.60 (3926)**	**-**
	$[0, 10^{-05}]$	0.09 (6290)	0.1 (3050)	0.12 (1664)	-
	$(10^{-05}, 10^{-04}]$	0.16 (4760)	0.18 (976)	0.2 (182)	-
	$(10^{-04}, 10^{-03}]$	0.36 (6290)	0.4 (2318)	0.41 (1040)	-
	$(10^{-03}, 1]$	0.76 (8330)	0.91 (2867)	1.09 (1040)	-

Table S4. Standard deviation (n pairs) of additive changes in relative abundance by study, rarefaction procedure, and average relative abundance category for each taxon.

		**Rarefaction (Percent of Study Minimum Read Count)**			
**Study**	**Taxon Abundance**	**None**	**100%**	**80%**	**60%**
**Moving Pictures (Gut)**	**All**	**0.00291 (309,036)**	**0.00290 (309,036)**	**0.00291 (309,036)**	**0.00292 (309,036)**
	[0, 10^{-05}]	1.2×10^{-05} (241,176)	1.5×10^{-05} (241,176)	1.6×10^{-05} (241,020)	1.7×10^{-05} (239,928)
	(10^{-05}, 10^{-04}]	0.000138 (41,418)	0.000142 (41,574)	0.000145 (41,184)	0.000153 (42,510)
	(10^{-04}, 10^{-03}]	0.00127 (18,408)	0.00128 (18,330)	0.00128 (18,954)	0.00129 (18,564)
	(10^{-03} ,1]	0.0179 (8034)	0.0180 (7956)	0.0181 (7878)	0.0180 (8034)
**Student Microbiome Project (Gut)**	**All**	**0.0133 (234,472)**	**0.0132 (234,472)**	**0.0133 (234,472)**	**0.0133 (234,472)**
	[0, 10^{-05}]	3×10^{-05} (171,402)	3.2×10^{-05} (171,031)	3×10^{-05} (170,660)	3×10^{-05} (171,402)
	(10^{-05}, 10^{-04}]	0.000586 (24,486)	0.000606 (24,486)	0.000573 (25,228)	0.000606 (24,486)
	(10^{-04}, 10^{-03}]	0.00562 (16,324)	0.00555 (16,695)	0.00558 (16,324)	0.00562 (16,324)
	(10^{-03} ,1]	0.0427 (22,260)	0.0427 (22,260)	0.0428 (22,260)	0.0428 (22,260)
**Gajer (Vaginal)**	**All**	**0.0239 (147,510)**	**0.0241 (147,510)**	**0.0239 (147,510)**	**0.0240 (147,510)**
	[0, 10^{-05}]	6.1×10^{-05} (72,861)	7.4×10^{-05} (71,073)	9.7×10^{-05} (75,990)	8.6×10^{-05} (70,179)
	(10^{-05}, 10^{-04}]	0.000631 (31,290)	0.000629 (33,525)	0.000660 (27,714)	0.000703 (33,525)
	(10^{-04}, 10^{-03}]	0.00537 (26,820)	0.00542 (26,373)	0.00522 (26,820)	0.00557 (26,373)
	(10^{-03} ,1]	0.0709 (16,539)	0.0714 (16,539)	0.0701 (16,986)	0.0695 (17,433)
**Ravel (Vaginal)**	**All**	**0.0367 (3926)**	**0.0368 (3926)**	**0.0372 (3926)**	**0.0369 (3926)**
	[0, 10^{-05}]	0 (1456)	0 (1664)	0 (1664)	0 (1820)
	(10^{-05}, 10^{-04}]	0.000393 (468)	0.000493 (182)	0.000679 (286)	NA (0)
	(10^{-04}, 10^{-03}]	0.00202 (910)	0.00240 (1040)	0.00237 (884)	0.00215 (936)
	(10^{-03} ,1]	0.0696 (1092)	0.0716 (1040)	0.0706 (1092)	0.0676 (1170)

Table S5. Standard deviation (n pairs) of multiplicative changes between consecutive nonzero relative abundances by study, rarefaction procedure, and average relative abundance category for each taxon.

		Rarefaction (Percent of Study Minimum Read Count)			
**Study**	**Taxon Abundance**	**None**	**100%**	**80%**	**60%**
**Moving Pictures (Gut)**	**All**	**1.17 (27,180)**	**1.12 (19,317)**	**1.1 (17,606)**	**1.09 (15,572)**
	[0, 10^{-05}]	0.62 (1304)	0.45 (379)	0.41 (217)	0.30 (115)
	(10^{-05}, 10^{-04}]	0.98 (9223)	0.83 (4621)	0.80 (3538)	0.74 (2615)
	(10^{-04}, 10^{-03}]	1.24 (10524)	1.15 (8486)	1.12 (8153)	1.09 (7107)
	(10^{-03} ,1]	1.38 (6129)	1.30 (5831)	1.25 (5698)	1.24 (5735)
**Student Microbiome Project (Gut)**	**All**	**1.84 (21,925)**	**1.68 (17,074)**	**1.65 (16,451)**	**1.63 (15,525)**
	[0, 10^{-05}]	1.13 (403)	0.97 (109)	0.74 (65)	0.84 (53)
	(10^{-05}, 10^{-04}]	1.45 (1821)	1.30 (723)	1.21 (728)	1.14 (565)
	(10^{-04}, 10^{-03}]	1.71 (4440)	1.48 (2801)	1.47 (2499)	1.44 (2165)
	(10^{-03} ,1]	1.93 (15,261)	1.74 (13,441)	1.7 (13,159)	1.68 (12,742)
**Gajer (Vaginal)**	**All**	**1.78 (4992)**	**1.52 (3112)**	**1.47 (2852)**	**1.39 (2644)**
	[0, 10^{-05}]	0.96 (29)	NA (0)	NA (1)	NA (0)
	(10^{-05}, 10^{-04}]	1.30 (182)	0.80 (34)	0.71 (37)	0.65 (17)
	(10^{-04}, 10^{-03}]	1.56 (1007)	1.23 (467)	1.28 (359)	1.08 (294)
	(10^{-03} ,1]	1.85 (3774)	1.57 (2611)	1.50 (2455)	1.43 (2333)
**Ravel (Vaginal)**	**All**	**1.54 (386)**	**1.4 (264)**	**1.39 (234)**	**1.41 (222)**
	[0, 10^{-05}]	NA (0)	NA (0)	NA (0)	NA (0)
	(10^{-05}, 10^{-04}]	0.09 (3)	NA (0)	NA (0)	NA (0)
	(10^{-04}, 10^{-03}]	1.09 (50)	0.83 (22)	1.04 (11)	0.85 (11)
	(10^{-03} ,1]	1.60 (333)	1.44 (242)	1.40 (223)	1.44 (211)

Table S6. Standard deviation (n pairs) of differences in CLR-transformed abundances between consecutive time points by study, rarefaction procedure, and average relative abundance category for each taxon.

		**Rarefaction (Percent of Study Minimum Read Count)**			
**Study**	**Taxon Abundance**	**None**	**100%**	**80%**	**60%**
**Moving Pictures (Gut)**	**All**	**0.48 (309,036)**	**0.40 (309,036)**	**0.38 (309,036)**	**0.35 (309,036)**
	$[0, 10^{-05}]$	0.20 (241,176)	0.13 (241,176)	0.12 (241,020)	0.10 (239,928)
	$(10^{-05}, 10^{-04}]$	0.73 (41,418)	0.56 (41,574)	0.52 (41,184)	0.47 (42,510)
	$(10^{-04}, 10^{-03}]$	1.14 (18,408)	0.99 (18,330)	0.95 (18,954)	0.90 (18,564)
	$(10^{-03}, 1]$	1.39 (8034)	1.29 (7956)	1.26 (7878)	1.22 (8034)
**Student Microbiome Project (Gut)**	**All**	**0.69 (234,472)**	**0.57 (234,472)**	**0.55 (234,472)**	**0.53 (234,472)**
	$[0, 10^{-05}]$	0.19 (171,402)	0.11 (171,031)	0.10 (170,660)	0.09 (171,402)
	$(10^{-05}, 10^{-04}]$	0.70 (24,486)	0.47 (24,486)	0.44 (25,228)	0.40 (24,486)
	$(10^{-04}, 10^{-03}]$	1.18 (16,324)	0.91 (16,695)	0.87 (16,324)	0.81 (16,324)
	$(10^{-03}, 1]$	1.78 (22,260)	1.57 (22,260)	1.54 (22,260)	1.49 (22,260)
**Gajer (Vaginal)**	**All**	**0.49 (147,510)**	**0.35 (147,510)**	**0.33 (147,510)**	**0.30 (147,510)**
	$[0, 10^{-05}]$	0.09 (72,861)	0.05 (71,073)	0.04 (75,990)	0.03 (70,179)
	$(10^{-05}, 10^{-04}]$	0.24 (31,290)	0.13 (33,525)	0.12 (27,714)	0.10 (33,525)
	$(10^{-04}, 10^{-03}]$	0.52 (26,820)	0.33 (26,373)	0.30 (26,820)	0.27 (26,373)
	$(10^{-03}, 1]$	1.24 (16,539)	0.92 (16,539)	0.87 (16,986)	0.81 (17,433)
**Ravel (Vaginal)**	**All**	**1.03 (3926)**	**0.60 (3926)**	**0.57 (3926)**	**0.52 (3926)**
	$[0, 10^{-05}]$	0.31 (1456)	0.12 (1664)	0.11 (1664)	0.08 (1820)
	$(10^{-05}, 10^{-04}]$	0.54 (468)	0.20 (182)	0.21 (286)	NA (0)
	$(10^{-04}, 10^{-03}]$	0.91 (910)	0.41 (1040)	0.37 (884)	0.30 (936)
	$(10^{-03}, 1]$	1.70 (1092)	1.09 (1040)	1.02 (1092)	0.91 (1170)
